# Sexual Dimorphism in the Brain Correlates of Adult-Onset Depression: A Pilot Structural and Functional 3T MRI Study

**DOI:** 10.3389/fpsyt.2021.683912

**Published:** 2022-01-05

**Authors:** Maria Chiara Piani, Eleonora Maggioni, Giuseppe Delvecchio, Adele Ferro, Davide Gritti, Sara M. Pozzoli, Elisa Fontana, Paolo Enrico, Claudia M. Cinnante, Fabio M. Triulzi, Jeffrey A. Stanley, Elena Battaglioli, Paolo Brambilla

**Affiliations:** ^1^Department of Pathophysiology and Transplantation, University of Milan, Milan, Italy; ^2^Department of Neurosciences and Mental Health, Fondazione Istituto di Ricovero e Cura a Carattere Scientifico (IRCCS) Ca' Granda, Ospedale Maggiore Policlinico, Milan, Italy; ^3^Neuroradiology Unit, Fondazione IRCCS Ca' Granda, Ospedale Maggiore Policlinico, Milan, Italy; ^4^Department of Psychiatry and Behavioral Neurosciences, Wayne State University School of Medicine, Detroit, MI, United States; ^5^Department of Medical Biotechnology and Translational Medicine, Università degli Studi di Milano, Segrate, Italy

**Keywords:** major depressive disorder, sex differences, brain morphology, brain function, inhibitory control, adult-onset depression

## Abstract

Major Depressive Disorder (MDD) is a disabling illness affecting more than 5% of the elderly population. Higher female prevalence and sex-specific symptomatology have been observed, suggesting that biologically-determined dimensions might affect the disease onset and outcome. Rumination and executive dysfunction characterize adult-onset MDD, but sex differences in these domains and in the related brain mechanisms are still largely unexplored. The present pilot study aimed to explore any interactions between adult-onset MDD and sex on brain morphology and brain function during a Go/No-Go paradigm. We hypothesized to detect diagnosis by sex effects on brain regions involved in self-referential processes and cognitive control. Twenty-four subjects, 12 healthy (HC) (mean age 68.7 y, 7 females and 5 males) and 12 affected by adult-onset MDD (mean age 66.5 y, 5 females and 7 males), underwent clinical evaluations and a 3T magnetic resonance imaging (MRI) session. Diagnosis and diagnosis by sex effects were assessed on regional gray matter (GM) volumes and task-related functional MRI (fMRI) activations. The GM volume analyses showed diagnosis effects in left mid frontal cortex (*p* < 0.01), and diagnosis by sex effects in orbitofrontal, olfactory, and calcarine regions (*p* < 0.05). The Go/No-Go fMRI analyses showed MDD effects on fMRI activations in left precuneus and right lingual gyrus, and diagnosis by sex effects on fMRI activations in right parahippocampal gyrus and right calcarine cortex (*p* < 0.001, ≥ 40 voxels). Our exploratory results suggest the presence of sex-specific brain correlates of adult-onset MDD–especially in regions involved in attention processing and in the brain default mode–potentially supporting cognitive and symptom differences between sexes.

## Introduction

Major depressive disorder (MDD) is a disabling psychiatric illness affecting an increasing proportion of the global population, reaching a peak of 5.74% in late adulthood, higher among females (6.75%) compared to males (4.60%) (1, 2).

The growing burden of disease in the elderly population is partly attributable to social risk factors, including stressful life events, which have been shown to facilitate MDD onset during adulthood (3–7). Of note, adult-onset MDD seems to have worse prognosis than MDD occurring at younger ages, as findings suggest that the course of illness is more severe, the recurrence rate is higher as well as is the suicide rate, which appears to be the 13th cause of death in this clinical group (3, 8–11). Executive dysfunction–including aberrant interference resolution and inhibitory control–is another core trait of late life depression, suggesting a cumulative burden of disease (12).

The higher prevalence of adult-onset MDD in females relative to males is in line with the documented sex gap in lifetime MDD rates (4, 13) and supports sex-specific models of MDD vulnerability, neurobiology, and psychopathology (14).

Notably, in MDD in general, sex differences are not limited to the rates of disease, but also involve its clinical presentation, with females developing more somatic symptoms, comorbid anxiety disorders and atypical depression, and males having higher mortality rates, both from suicide and from somatic comorbidity or substance abuse (4, 5, 15). Differences in the cognitive correlates of MDD between females and males have also been observed, even if the results appear fragmented (16, 17).

Up to now, fewer studies have attempted to disambiguate the role of sex in adult-onset MDD (18–20), leaving a number of questions either undiscussed or unresolved. Of note, sex differences in disease susceptibility do not appear to be merely due to sex-specific responsivity to stressors (7).

Overall, a more complex interplay among genetic loading, stress susceptibility, and life events might contribute to sex-specific risk factors for adult-onset MDD, which in turn might result in neurobiological and clinical features that are at least partly different between sexes.

Nevertheless, most of the available knowledge on the topic regards MDD in general. From a neurobiological perspective, like in healthy individuals who show numerous sex-related brain differences throughout the lifespan (21–25), there is evidence of peculiar patterns of brain alterations in males and females affected by MDD (26). Sex-specific deficits in GM volume have been observed in regions within the frontal and temporal cortices and the prefrontal-striatal circuit in MDD males, and in areas of the prefrontal-limbic circuit and the lingual gyrus in MDD females (27–30). Interaction effects of late life depression and sex on frontal regional brain volumes were suggested as well (19).

Functional Magnetic Resonance Imaging (fMRI) paradigms in MDD reported sex differences in the fMRI correlates of facial emotion processing and autobiographical memory recall (31, 32).

Despite the wide profile of cognitive dysfunctions characterizing MDD, sex differences in cognitive performances and in the underlying functional brain networks remain largely unexplored. The cognitive domain of inhibitory control is receiving special interest, since it appears to be greatly affected by the MDD pathology and to impact on the therapeutical and functional outcomes (33–37).

This domain is often assessed with the Go/No-Go paradigm, which is a well-established tool also used to measure sustained attention (38, 39). Adapted versions of the Go/No-Go task, especially those with affective valences, have been used to study brain activations in MDD patients compared to healthy controls, but evidence of sexual dimorphisms remains scarce. On this regard, Chuang et al. (40) reported group by sex effects on the fMRI response to the affective task in the supramarginal gyrus and in the posterior cingulate cortex (PCC), but not on task performances (40). Additionally, van Deurzen et al. (41) proposed that inhibitory control impairments may predict the onset of affective disorders, including depression, in adolescent females (41). Moreover, evidence from preclinical studies points at a possible sexual dimorphism in the mechanisms that underlie stress-induced alterations in structural brain plasticity and sustained attention disruption in MDD (41).

Sex dimorphisms in inhibitory control have been reported, but mostly in healthy individuals. Concerning the Go/No-Go paradigm, females showed slightly lower accuracy while processing the Go vs. No-Go contrast, and also longer processing times for the monitoring of response conflict, motor response execution, and outcome inhibition (42, 43). In addition, concerning patterns of brain activation, it has been shown that, during impulse inhibition, males tend to show more activity in the anterior cingulate cortex (ACC), while females in the middle temporal cortex (44). Conversely, sex differences in pure inhibitory control and related brain mechanisms are more poorly documented in MDD. In fact, the only evidence available indicates that deficits in this domain may predict the onset of affective disorders, including depression, in adolescent females (41). Adapted versions of the Go/No-Go task, especially those with affective valences, have been used to study the neural bases of emotion-cognitive interference in MDD. On this regard, Chuang et al. (40) reported group by sex effects on the fMRI response to the affective task in the supramarginal gyrus and in the posterior cingulate cortex (PCC), but not on task performances (40). Of note, evidence from preclinical studies points at a possible sexual dimorphism in the mechanisms that underlie stress-induced alterations in structural brain plasticity and sustained attention disruption in MDD (41).

To summarize, despite the remarkable sex differences in MDD and the increasing evidence that its clinical heterogeneity is biologically-determined, the impact of sex on MDD clinical presentation and outcome and on the underlying brain processes has been often underestimated and remains unclear (45).

In this context, very little is still known about sex-related brain mechanisms responsible for inhibitory control and sustained attention in adult-onset MDD. Investigating sexual dimorphisms in these domains may be particularly important for choosing the most appropriate treatment plan, since the functional connectivity of the cognitive control network has been demonstrated to be a good predictor of antidepressant treatment response and to be as well correlated to symptom improvement (46).

On account of these premises, the present work aimed to preliminarily explore the impact of sex and adult-onset MDD on brain morphology and function, especially in the brain regions involved in response inhibition and sustained attention. To this end, we carried out a pilot 3T structural and functional MRI study on subjects with adult-onset MDD and healthy controls and assessed the putative role of sex on brain changes in MDD, in terms of both regional GM volumes and activation patterns during a standard parametric Go/No-Go task.

## Materials and Methods

### Study Population

Twenty-four subjects, 12 affected by MDD and 12 healthy controls (HC), aged between 50 and 90 years were enrolled in the study. Clinical diagnoses were assessed using the Italian version of the Structured Clinical Interview for DSM-5 (SCID): only subjects with adult-onset MDD (≥ 45 years) were included in the patient group, and only subjects without psychiatric diagnoses were included in the control group. Exclusion criteria for patients were lifetime psychotic symptoms and current DSM-5 comorbid disorders. Exclusion criteria for all participants comprised lifetime alcohol or substance abuse, intellectual disability, history of head trauma with loss of consciousness, neurological or neurodegenerative illnesses.

All subjects provided a written informed consent to the study protocol, which was conducted in accordance with the Declaration of Helsinki and approved by the Ethical Committee of the Fondazione IRCCS Ca' Granda Ospedale Maggiore Policlinico, Milan, Italy.

### Psychopathological and Cognitive Assessment

In MDD patients, information on age of onset and current pharmacological treatment were collected. All participants underwent a comprehensive psychopathological assessment including the Hamilton Depression Rating Scale (HDRS) (47), the Depression Anxiety and Stress Scales-21 (DASS-21) (48), and the Traumatic Experience Checklist (TEC) (49). The participants' cognitive performances were rated using the Repeatable Battery for the Assessment of Neuropsychological Status (RBANS) (50).

### Multimodal Neuroimaging Acquisition

Structural and functional MRI data were acquired on a 3T Philips Achieva DStream scanner (Philips, Best, The Netherlands) equipped with a 32-channel head coil. Structural T1-weighted images were collected using a 3D turbo field echo (TFE) SENSE sequence (field of view: 250 mm (FH) × 240 mm (AP) × 180 mm (RL), voxel size: 1 mm^3^, echo time (TE): 4 ms, repetition time (TR): 8 ms, flip angle: 8°). Two hundred (plus two dummy) functional MRI volumes were collected during a visuomotor task (described in section the following) using a multi-transmit T2^*^-weighted echo planar imaging (EPI) sequence (field of view: 120 mm (FH) × 256 mm (AP) × 256 mm (RL), voxel size: 2 × 2 × 2 mm, echo time (TE): 30 ms, repetition time (TR): 2,000 ms, flip angle: 90°). Five additional (plus two dummy) fMRI scans acquired with the same parameters but opposite phase encoding direction were used to estimate and correct for susceptibility induced distortions in the fMRI volumes.

#### fMRI Experimental Protocol

The fMRI experiment was a visuomotor Go/No-Go task implemented using the Presentation® software (Neurobehavioral Systems, Inc., Berkeley, CA, USA). Visual stimuli were delivered in the MR scanner through the MR-compatible NordicNeuroLab VisualSystem HD (NNL, Bergen, Norway) and synchronized to the fMRI pulses using the NNL SyncBox. The participants' responses were collected using the MR-compatible NNL ResponseGrip device (composed of thumb and index finger buttons). Prior to the fMRI session, all subjects were trained to perform the task outside the MRI environment.

The Go/No-Go task combined blocks with only target stimuli (“Go” blocks) with blocks with both target and non-target stimuli (“Go/No-Go” blocks). Consecutive stimuli were delivered either at 1 s intervals (“Periodic” blocks) or at random intervals around 1 s (“Random” blocks). In the “Go” blocks, the subject was asked to push the thumb button every time a red or green flashing square appeared on the screen (excitation mode). In the “Go/No-Go” blocks, the participants were asked to push every time a green square flashed on the screen, and to not push when a red square flashed on the screen (inhibition mode). All responses were collected using the dominant thumb, which was in the right hand for all subjects. The experiment consisted of eight task blocks (two “Periodic Go”, two “Periodic Go/No-Go”, two “Random Go”, two “Random Go/No-Go”, lasting 32 s each) alternated with resting state 16 s intervals, during which the subjects had to focus on a fixation cross at the center of the screen and to not think of anything specific.

### Data Processing

The processing of clinical, behavioral, and neuroimaging data was performed on Matlab R2019a Update 4 (The Mathworks, Inc.). Neuroimaging analyses were conducted using the Matlab-based Statistical Parametric Mapping (SPM) software (version 12, https://www.fil.ion.ucl.ac.uk/spm) (51), its CAT12 toolbox (http://141.35.69.218/cat/) (52), and Matlab in-house scripts. The FMRIB Software Library (FSL) was also used for fMRI pre-processing (53). Statistical comparisons were performed using SPM12 statistical tools and the Statistics and Machine Learning Toolbox™.

#### Extraction of Brain Morphological Features

##### Pre-processing

The participants' T1-weighted images were visually inspected and manually reoriented as the SPM tissue probability maps (TPMs). Brain tissue segmentation was performed using the CAT12 segmentation tool. The pipeline included the SPM bias inhomogeneity correction, initial affine registration to SPM12 TPMs, SPM unified segmentation, skull stripping, local adaptive segmentation, adaptive maximum a posterior (AMAP) segmentation, and optimized shooting registration to the CAT12 default template in the Montreal Neurological Institute (MNI) space. Spatially normalized and modulated GM images were resampled (voxel size: 1.5 × 1.5 × 1.5 mm) and smoothed with a 3D 6 mm Full Width Half Maximum (FWHM) Gaussian kernel. Total intracranial volumes (ITV) were extracted from the subjects' CAT12 report files. The processing quality was checked by displaying the normalized and modulated GM images (one slice per subject) and by checking their correlation among subjects to identify outliers. The quality of the pre-processed GM maps was reasonable for all subjects; therefore no subjects were excluded.

##### Region-Based Volume Estimation

Region of interest (ROI) analyses were performed using in-house Matlab scripts. The normalized, modulated, and smoothed GM images were used to extract region of interest (ROI) GM volumes in the 116 Automated Anatomical Labeling (AAL) atlas regions (54).

#### Extraction of Brain Functional Features

##### Pre-processing

For each subject, the pre-processing of the fMRI volumes recorded during the Go/No-Go task was performed using SPM12 and FSL tools. In SPM12, head motion artifacts were estimated and used to realign all fMRI volumes to the first reference fMRI volume. For all but one subjects, the movement parameters felt within the pre-specified limits (translation < 2 mm, rotation < 3°). The realigned fMRI volumes (and the additional fMRI data with inverse phase-encoding polarity) were imported in FSL, in which the TOPUP tool was used to estimate and correct for susceptibility-induced distortions. This correction step could not be applied to the fMRI volumes from two subjects due to the absence of the inverse polarity data. In SPM12, the subject's structural image was co-registered to the mean fMRI image and used to estimate the deformation fields for spatial normalization. The fMRI volumes were then normalized to the MNI space and spatially smoothed using a 3D 4 mm FWHM Gaussian kernel. The post-processing fMRI data quality in the subject with 3.7 mm translation was carefully verified and considered sufficient for inclusion in the analyses.

##### Subject-Level fMRI Statistics

The pre-processed fMRI volumes from each subject were entered in a voxel-based block-related GLM activation analysis. The BOLD signal from each voxel was modeled as a function of both task-related and confounding factors (regressors). The GLM design matrix included one regressor for each experimental condition (1. “Periodic Go”, 2. “Periodic Go/No-Go”, 3. “Random Go”, 4. “Random Go/No-Go”) obtained by the block convolution with the canonical hemodynamic response function (HRF), and head translation (n=3) and rotation (n=3) parameters as confounding regressors.

After estimation of the GLM coefficients, inference on the experimental effects was performed with appropriate *t-*contrasts (*t-*tests on linear combinations of the coefficients). The following subject-level *t-*contrasts were considered for the group-level statistics: 1. task *vs*. rest, 2. “Go” *vs*. rest, 3. “Go/No-Go” *vs*. rest, 4. “Go” *vs*. “Go/No-Go”.

#### Statistical Analyses

The statistical comparisons of demographic, clinical, cognitive, and brain morphological data were performed using in-house Matlab scripts based on the Statistics and Machine Learning Toolbox™ functions. The effects of diagnosis, sex, and their interaction on clinical (HDRS, DASS-21, TEC) and cognitive (RBANS, fMRI performance) measures were assessed using linear regression models where the selected variable was explained in terms of diagnosis and sex, in interaction, and age. Inference on the GLM coefficients was performed using two-sided *t-*tests, with significance threshold placed at *p* = 0.05.

In the brain morphological analyses, the AAL ROI volumes were used as dependent variables in univariate General Linear Model (GLM) designs, in which they were modeled as a function of diagnosis, sex, age, and ITV, with diagnosis and sex interacting. After GLM least-squares fitting estimation, diagnosis, sex, and diagnosis by sex effects were assessed via two-sided *t*-tests on the GLM coefficients. Significance threshold was set to *p* = 0.05 after multiple comparison correction using Bonferroni's method (n=116). Due to the conservativeness of such correction, tendencies are also reported (*p* < 0.01 for main factor effects, *p* < 0.05 for interaction effects).

Group-level statistics on the fMRI activations were extracted using SPM12 functions. The subject-level fMRI contrast maps were entered in second-level random-effects GLM analyses. For each contrast, we employed a full-factorial GLM design with diagnosis and sex factors in interaction, and age and “Go/No-Go” task performances (1. Percent of responses to target stimuli, 2. Time of response to target stimuli, 3. Percent of responses to non-target stimuli) as covariates of no interest. After GLM estimation, we assessed diagnosis, sex, and diagnosis by sex effects on the subject-level fMRI activations using positive and negative one-sided *t*-tests enabled by SPM12 (*p* < 0.001, > 40 voxels). The significant clusters were localized based on the AAL atlas and the subjects' peak contrast values were illustrated as a function of diagnosis, sex, or their combination, as appropriate.

## Results

### Demographic, Clinical, and Cognitive Information

The sample characteristics are summarized in Table 1. Further details on the patients' therapies and dosages and disease characteristics can be found in Supplementary Tables S2, S3. Sex distribution was comparable between MDD and HC groups. Age was comparable between MDD subjects and HC, females and males, and diagnosis by sex subsets. Total HDRS scores were higher in MDD subjects compared to HC and in females compared to males, but no diagnosis by sex effects emerged. Females also showed higher DASS-21 stress and depression scores than males, regardless of the diagnostic group. No effects of diagnosis, sex nor their interaction emerged on DASS-21 anxiety and TEC scores.

**Table 1:** Demographic, clinical, and cognitive information of the sample.

	**MDD**			**HC**			**Diagnosis (MDD vs. HC)**	**Sex (M vs. F)**	**Diagnosis by sex**
	**Total**	**F**	**M**	**Total**	**F**	**M**	**Stats**	**Stats**	**Stats**
Sex, N	12	5	7	12	7	5	χ^2^ = 0.67, *p =* 0.41	–	–
Age [years]	66,5 ± 9,1	64 ± 11,7	68,2 ± 7,2	68,7 ± 12,3	64,4 ± 13,1	74,6 ± 9	T = −0.07, *p =* 0.95	T = 1.65, *p =* 0.12	T = −0.67, *p =* 0.51
Medication, N^*^	3 SSRI, 2 SNRI, 2 BDZ, 1 AC, 1 lithium, 1 NSAID, 1 AAP	1 SSRI, 1 AC, 1 BDZ, 1 NSAID	2 SSRI, 2 SNRI, 1 BDZ, 1 lithium, 1 AAP	1 SSRI^∧^, 1 MAOI^§^	1 SSRI^∧^, 1 MAOI^§^	–	–	–	–
HDRS score	11 ± 6.5	13.8 ± 7.8	8.8 ± 4.9	3.7 ± 5.2	6.1 ± 5.7	0.2 ± 0.4	**T = 3.03**, ***p <*** **0.01**	**T = −3.35**, ***p <*** **0.01**	T = 0.78, *p =* 0.45
DASS−21 D score	13.2 ± 13.7	21.2 ± 17.8	7.4 ± 6.5	7.8 ± 9.5	11.7 ± 10.9	2.4 ± 2.6	T = 1.91, *p =* 0.07	**T = −2.84**, ***p =*** **0.01**	T = −0.12, *p =* 0.91
DASS−21 A score	8.5 ± 10.7	14.4 ± 12.9	4.3 ± 7.1	6.7 ± 8.1	8.6 ± 10.2	4.0 ± 3.2	T = 1.28, *p =* 0.22	T = −1.78, *p =* 0.09	T = −0.45, *p =* 0.66
DASS−21 S score	15.3 ± 14.2	25.6 ± 16.1	8.0 ± 6.4	13.1 ± 8.3	17.7 ± 5.8	6.8 ± 7.1	T = 1.47, *p =* 0.16	**T = −2.27**, ***p =*** **0.04**	T = −0.70, *p =* 0.49
TEC score	16 ± 10.1	16.2 ± 11.3	15.8 ± 10.1	13.6 ± 15.0	19.1 ± 18.0	5.8 ± 3.1	T = −0.39, *p =* 0.70	T = −1.93, *p =* 0.07	T = 1.33, *p =* 0.20
RBANS score	95.2 ± 15.3	94.2 ± 17.2	95.8 ± 15.2	95.3 ± 13.6	99.1 ± 13.5	90.0 ± 13.3	T = −0.74, *p =* 0.47	T = −0.10, *p =* 0.92	T = 0.58, *p =* 0.57
Target resp %	91.91 (8.06)	87.70 (9.26)	94.92 (6.05)	91.51 (8.10)	96.30 (3.30)	84.81 (8.23)	**T = −2.49**, ***p =*** **0.02**	**T = −2.22**, ***p =*** **0.04**	**T = 3.37**, ***p*** **<0.01**
Target resp time	3,365.486 (563.642)	3,723.803 (547.062)	3,109.545 (446.656)	3,222.856 (499.719)	3,242.857 (401.313)	3,194.545 (665.936)	**T = 2.39**, ***p =*** **0.02**	T = −1.83, *p =* 0.08	T = −1.21, *p =* 0.24
Non–target resp %	31.000 (12.548)	27.200 (3.347)	33.714 (15.142)	27.667 (19.704)	21.143 (14.554)	36.800 (23.900)	T = 0.64, *p =* 0.53	T = 1.39, *p =* 0.18	T = −0.61, *p =* 0.55
Non–target resp time [ms]	3,282.353 (716.570)	3,529.601 (447.813)	3,105.748 (848.731)	2,959.744 (1,118. 360)	3,107.667 (1,219.406)	2,752.653 (1,056.965)	T = 0.80, *p =* 0.43	T = −1.16, *p =* 0.26	T = 0.15, *p =* 0.89
Go resp %	96.653 (2.948)	95.118 (3.833)	97.750 (1.666)	89.239 (21.420)	96.175 (6.072)	79.527 (31.692)	T = −0.11, *p =* 0.91	T = −1.90, *p =* 0.07	T = 1.58, *p =* 0.13
Go resp time [ms]	2,856.840 (482.479)	3,128.650 (540.870)	2,662.691 (355.251)	2,943.950 (473.858)	2,910.402 (377.190)	2,990.917 (631.943)	T = 0.94, *p =* 0.36	T = −0.58, *p =* 0.57	T = −1.20, *p =* 0.25

*For continuous variables, mean ± standard deviations are reported. F, females; M, males. MDD, major depressive disorder, HC, healthy controls; T, T-statistics; χ^2^, χ^2^ statistics; p, p-value. Group, sex, and group by sex effects were assessed using linear regression models and one-sided t-tests on their coefficients. SSRI, selective serotonin reuptake inhibitors; SNRI, selective serotonin and norepinephrine reuptake inhibitors; BDZ, benzodiazepines; AAP, atypical antipsychotics; AC, anticonvulsants; NSAID, nonsteroidal anti-inflammatory drugs; MAOI, monoamine oxidase inhibitors; HDRS, Hamilton depression rating scale; DASS-21, depression anxiety stress scale, 21 items (D, depression; A, anxiety; S, stress); TEC, traumatic experiences checklist; RBANS, repeatable battery for the assessment of neuropsychological status*.

∧ *Paroxetine 20 mg for medical conditions*.

§ * Amitriptyline 16 mg for medical conditions*.

* *The data from one female adult-onset MDD are not available. Significant T statistics are highlighted in bold*.

Regarding cognitive performances, total RBANS scores were comparable across diagnosis and sex groups, whereas the fMRI task performances were more influenced by these factors. In the “Go/No-Go” blocks (inhibition mode), the percentage of correct responses to target stimuli (hits) was slightly higher in HC compared to MDD and in females compared to males, with stronger diagnosis by sex effects. Specifically, hit rates were higher in healthy females than in healthy males, lower in MDD females than in MDD males. The times of response to target stimuli were higher in MDD patients than in HC, but no sex or group by sex effects emerged. No influences of diagnosis and sex emerged on the percentage of commission errors (i.e., responses to non-target stimuli) and on the delay of such incorrect responses. Similarly, the performance rates in the “Go” blocks (excitation mode) were comparable across diagnoses and sexes.

### Diagnosis and Sex Effects on Brain ROI Morphology

**Figure 1:**
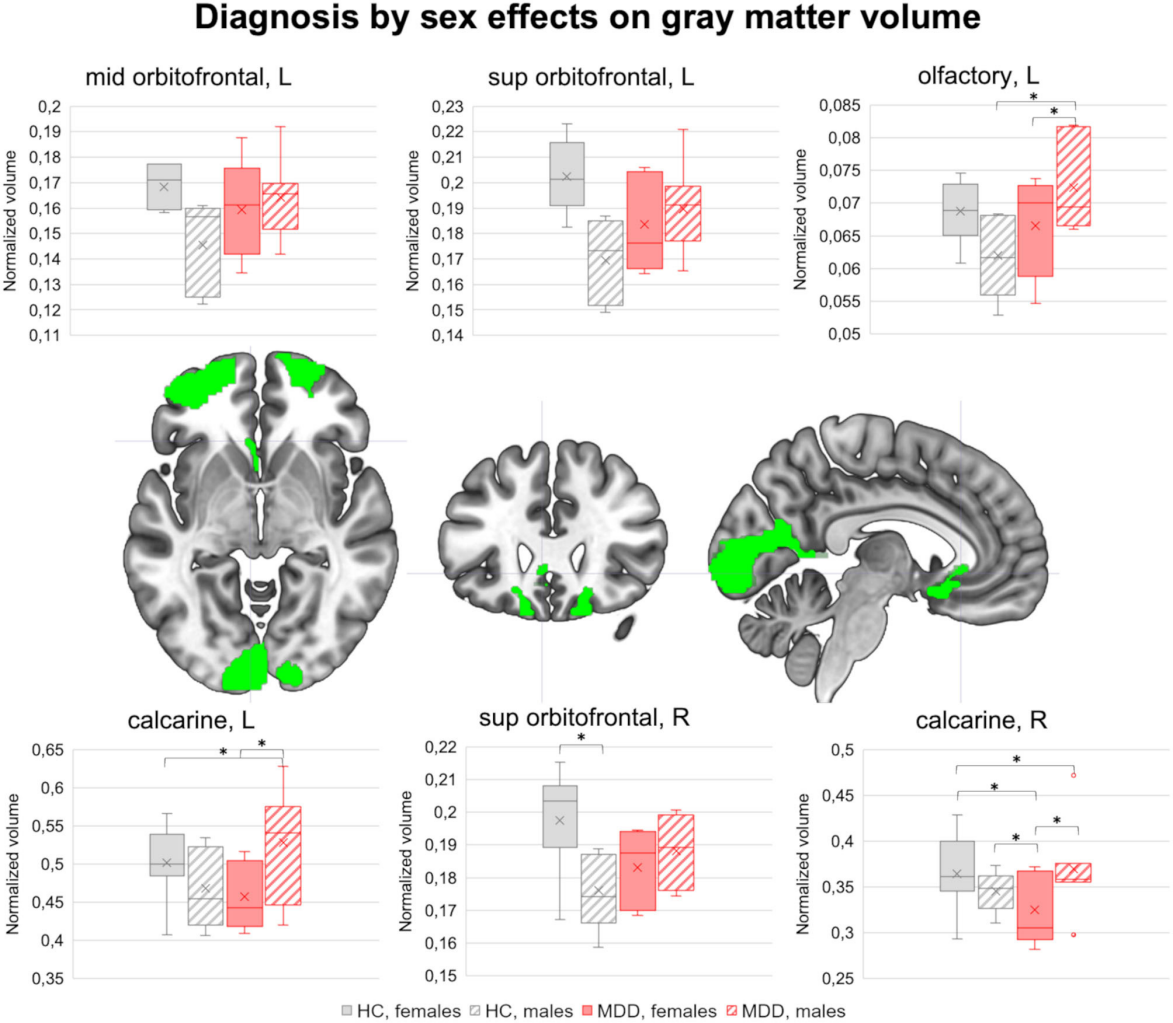
Diagnosis by sex effects on brain morphology. The brain clusters showing sexually dimorphic GM volume differences between MDD and HC groups are highlighted in green (*p* < 0.05). In the top and bottom rows, the boxplots represent the normalized GM volume distribution in HC females, HC males, MDD females, and MDD males. Pairwise differences between diagnosis by sex subsets (net of age and ITV) are highlighted (*p* < 0.05). GM, gray matter; MDD, major depressive disorder; HC, healthy controls; L, left; R, right. ^*^significant pairwise differences (*p* < 0.05).

Regional GM volumes in the AAL ROIs were comparable between MDD and HC groups, females and males, and diagnosis by sex subsets (*p* < 0.05, Bonferroni corrected), but some tendencies emerged. Compared to HC, MDD subjects showed lower GM volume in the left mid frontal cortex (T = 3.19, *p* = 0.005). Diagnosis by sex effects emerged also in the orbitofrontal gyrus [left middle (T = 2.18, *p* = 0.043) and superior (T = 2.79, *p* = 0.012) portions, right superior portion (T = 2.25, *p* = 0.037)], in the left olfactory cortex (T = 2.56, *p* = 0.019), and in the bilateral calcarine cortex (left: T = 2.30, *p* = 0.033; right: T = 2.21, *p* = 0.040). As shown in Figure 1, the normalized GM volume in all these ROIs was higher in healthy females than in healthy males, but lower in MDD females compared to MDD males. The pairwise differences between group by sex subsets are marked in the boxplots (*p* < 0.05).

### Diagnosis and Sex Effects on Task-Related fMRI Activations

**Figure 2:**
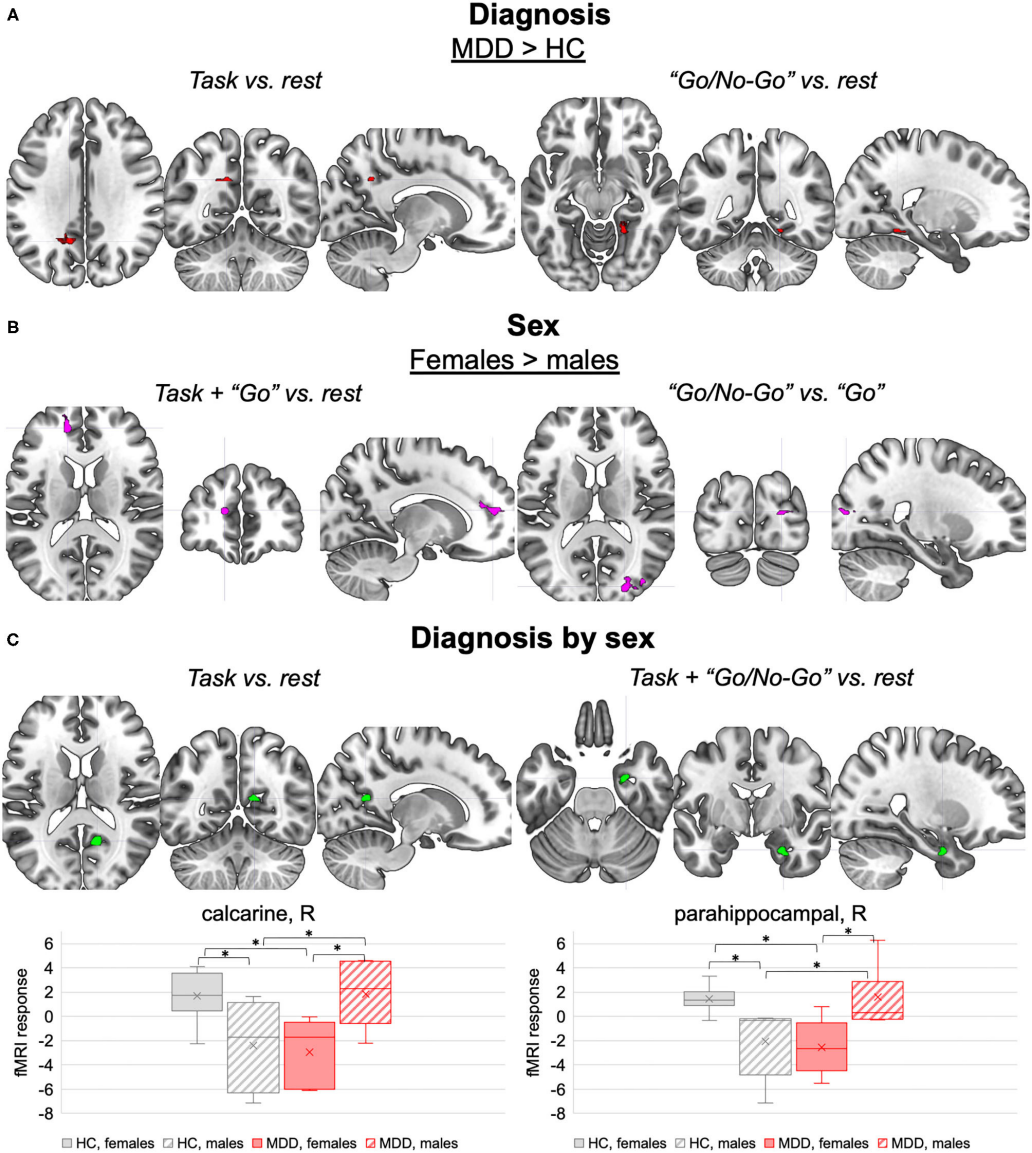
Diagnosis, sex, and interaction effects on task-related fMRI activations. **(A)** Diagnosis effects. Brain clusters with different fMRI responses to the task (left panel) and to the “Go/No-Go” inhibitory blocks (right panel) in MDD compared to HC (*p* < 0.001, ≥ 40 voxels). **(B)** Sex effects. Brain clusters with different fMRI responses to the task and “Go” excitatory blocks (left panel), and the “Go/No-Go” inhibitory blocks vs. “Go” excitatory blocks (right panel) in females compared to males (*p* < 0.001, ≥ 40 voxels). **(C)** Diagnosis by sex effects. Brain clusters with interaction effects on fMRI responses to the task (left panel) and the “Go/No-Go” inhibitory blocks (right panel) (*p* < 0.001, ≥ 40 voxels). The boxplots in the lower panel represent the corresponding normalized peak fMRI response distributions in HC females, HC males, MDD females, and MDD males. Pairwise differences in the peak fMRI response between diagnosis by sex subsets (net of age) are highlighted (*p* < 0.05). MDD, major depressive disorder; HC, healthy controls; L, left; R, right. *significant pairwise differences (*p* < 0.05).

This section is dedicated to the effects of diagnosis and sex on the fMRI activations induced by the Go/No-Go task, which are detailed in Table 2 and illustrated in Figure 2. The fMRI activations emerged in the entire group, described in the Supplementary Material, are used as reference to interpret the influences of diagnosis and sex.

**Table 2:** Diagnosis and sex modulations on the task-related fMRI activations.

**Effect**	**Contrast**	**# voxels**	**x, y, z**	**AAL region**	**T–stat**	***p***
diagnosis (MDD vs. HC)	task vs. rest	40	−12, −56, 34	Precuneus, L	4.84	<0.001
	“Go/No–Go” vs. rest	46	22, −52, −8	Lingual gyrus, R	4.67	<0.001
sex (females vs. males)	task vs. rest	76	−14, 46, 14	Anterior cingulate gyrus, L	5.81	<0.001
	“Go” vs. rest	40	−14, 48, 16	Superior frontal gyrus, medial L	5.35	<0.001
	“Go” vs. “Go/No–Go”	70	40, −82, 14	Middle occipital gyrus, R	5.54	<0.001
diagnosis by sex	task vs. rest	46	24, −4, 28	Parahippocampal gyrus, R	7.39	<0.001
		50	14, −60, 14	Calcarine cortex, R	6.06	<0.001
	“Go/No–Go” vs. rest	48	24, −4, −28	Parahippocampal gyrus, R	7.32	<0.001

*AAL, automated anatomical labeling atlas; T, T-statistics; p, p-value; x,y,z, MNI coordinates expressed in mm; MDD, major depressive disorder; HC, healthy controls; L, left; R, right*.

#### Diagnosis Modulation on fMRI Activations

Significant effects of MDD diagnosis were observed on the fMRI response to the task in general and to the “Go/No-Go” condition (*p* < 0.001, ≥ 40 voxels) (Table 2, section “Main effect of diagnosis”, Figure 2A). Indeed, when compared to HC, MDD subjects showed higher fMRI response to the task (vs. rest) in a portion of the left precuneus, and higher fMRI response to the “Go/No-Go” condition (vs. rest) in a portion of the right lingual gyrus. Both clusters were proximal to DMN nodes with negative BOLD response to the task, suggesting that MDD subjects might fail to deactivate these regions. No differences between MDD and HC groups were observed in the fMRI response to the “Go” vs. rest and the “Go/No-Go” vs. “Go” conditions.

#### Sex Modulation on fMRI Activations

Females and males were characterized by different fMRI responses to the task in general and to both “Go” and “Go/No-Go” conditions (*p* < 0.001, ≥ 40 voxels) (Table 2, section “Main effect of sex”, Figure 2B). When compared to males, females showed enhanced fMRI activation during the task, especially in the “Go” condition, in a cluster in the left hemisphere at the interface between anterior cingulate cortex and superior medial frontal cortex. Females also showed higher fMRI response than males to “Go” vs. “Go/No-Go” conditions in a cluster in the right middle and superior occipital cortex. Conversely, the fMRI activations induced by the “Go/No-Go” condition were not influenced by sex.

#### Diagnosis by Sex Modulation on fMRI Activations

Interaction effects were observed in the fMRI responses to the task in general and to its “Go/No-Go” condition (*p* < 0.001, ≥ 40 voxels) (Table 2, section “Diagnosis by sex effects”, Figure 2C). The fMRI response to the task and to the “Go/No-Go” condition in a portion of the right parahippocampal gyrus showed diagnosis by sex effects. As illustrated in Figure 2C, such response was more negative in MDD females compared to MDD males, whereas an opposite tendency emerged in the HC group. Similar interaction effects were observed in the fMRI response to the task in a cluster in the right calcarine cortex, which was significantly deactivated by the task at the group level (see Supplementary Material). The fMRI activations concerning the “Go” vs. rest and “Go” vs. “Go/No-Go” conditions did not exhibit interaction effects.

## Discussion

The focus of this study was to explore brain morphology and functional activations during a Go/No-Go task in a pilot sample of adult-onset MDD patients and HC, while paying attention to possible differences between females and males. To our knowledge, this is the first attempt to assess sex differences in the brain morphological and cognitive-related functional features of adult-onset MDD. Despite being preliminary, the obtained results suggest reduced GM volume in adult-onset MDD compared to HC in the left mid frontal cortex, coupled with diagnosis by sex effects on GM volumes within the orbitofrontal gyrus, the left olfactory cortex, and the bilateral calcarine cortex. In the same line, the fMRI analyses showed interaction effects on the Go/No-Go activation patterns in the right parahippocampal gyrus and in the right calcarine cortex, as well as higher fMRI responses in the left precuneus and in the right lingual gyrus in adult-onset MDD compared to HC.

This pilot evidence, which needs to be reproduced on larger samples, confirms the importance of considering sex as a relevant neurobiological factor in studies on MDD, including adult-onset disease, paving the way for future patient-centered investigations. A better knowledge of sexual dimorphism in adult-onset MDD could be particularly helpful in predicting the disease vulnerability over lifetime and in understanding the differential sex response to the available antidepressant treatments (55), allowing the identification of new targets for personalized interventions.

### Diagnosis by Sex Effects on Brain Morphology

Overall, the majority of the areas emerged from the structural MRI comparison between adult-onset MDD and HC are involved in different aspects of emotional processing and regulation and included the left middle frontal cortex, the orbitofrontal cortex, and the left olfactory cortex. Additionally, selective deficits in the calcarine cortex, a region involved in diverse cognitive processes, were also observed.

#### Diagnosis Modulation on ROI Morphology

The structural MRI comparison showed that adult-onset MDD patients, regardless of sex, had lower GM volumes in the left mid frontal cortex, an area that in the healthy brain is known to have a key role in several cognitive (56–59) and emotional processes (60, 61). Notably, our result is in line with evidence from previous studies on MDD patients (30, 62, 63).

Moreover, in depression, reduced GM volumes (63) in this area have been associated with impaired attention, psychomotor retardation and cognitive impairments as well as with pathological negative affectivity in response to negative emotional stimuli (60, 61, 64). Therefore, our results together with previous evidence support the key role of this region in MDD and further highlight the putative role of this structure in the development of clinical and cognitive manifestations of MDD that involve both males and females.

#### Diagnosis by Sex Modulation on ROI Morphology

A diagnosis by sex interaction effect was observed in three different areas, including the orbitofrontal cortex, the olfactory cortex, and the calcarine cortex, where healthy females showed higher GM volumes compared to healthy males, whereas adult-onset MDD patients reported an opposite pattern.

With regards to the orbitofrontal cortex, this brain region is involved in the behavioral modulation through the integration of sensory and visceral motor information, and has a key role in emotional processing (65). Although some studies reported the presence of structural differences between healthy males and females in this area (24, 25, 66, 67), the results are not univocal, possibly due to the different study characteristics. In contrast, the GM reduction in the orbitofrontal cortex in MDD was already observed by our and other research groups (68–72). Some studies also reported a correlation between deficits in this area and illness severity (72–74). Therefore, the increased GM deficits in the orbitofrontal cortex observed in adult-onset MDD females may explain the higher rates of internalizing problems and rumination often observed in the female sex, which could also be explained by the presence of different illness-related genes that in turn contribute to the different symptomatology observed in males and females with MDD (14, 75–77).

The same pattern of diagnosis by sex effects was observed in the olfactory cortex. Besides its key role in olfaction, this structure was found to be involved in emotional regulation (78). The presence of physiological sexual dimorphism on the area is not surprising, since literature evidence reports sex-related differences in olfactory ability and related pathways (79, 80), although there is no specific evidence regarding the olfactory cortex itself (79, 81).

With regards to the opposite sexually dimorphic pattern observed in adult-onset MDD, there is evidence supporting the fact that in depression the hedonic features of olfaction are impaired together with the sensitivity and identification of odors (82), which are also correlated to the severity of disease and emotional dysregulation (83, 84), ultimately suggesting a role of this structure in the pathogenesis (83, 84). Therefore, based on this evidence, lower GM volumes observed in the olfactory cortex in female adult-onset MDD patients are possibly correlated to worse affectivity and emotional dysregulation compared to male MDD patients.

Finally, healthy females showed higher GM volumes than healthy males in the calcarine cortex, a key region involved in processing visual information (85). Several studies reported the presence of different visuospatial information processing in the two sexes and of sex-related structural differences, although not consistently (86, 87). Notably, our results are in line with previous evidence that found higher GM volumes in females (88, 89), ultimately supporting the hypothesis that this structure can be considered a possible neuroanatomical classifier of biological sex. Conversely, sex-related differences in this area have not been previously observed in MDD. However, some evidence reported GM volume reduction and connectivity alterations in this area in MDD (90, 91), which were correlated to the presence of negative cognitive models (92, 93) and impairments in other cognitive domains, such as attention and working memory (94–96). Therefore, the reduced GM volumes observed in females with adult-onset MDD compared to their male counterpart may determine higher impairments in those cognitive domains, as preliminarily observed in our sample in relation to the fMRI task performances (hit rates). However, further studies are needed to corroborate this finding.

### Sex by Diagnosis Effects on Task-Related fMRI Activations

#### Diagnosis Modulation of fMRI Activations

Our results showed significant effects of adult-onset MDD diagnosis on fMRI response to the task in general and to the inhibitory condition, with adult-onset MDD patients showing reduced deactivation in the left precuneus and in the right lingual gyrus compared to HC.

Specifically, the precuneus is well known to be a functional core of the DMN (97, 98), which is a large-scale brain network encompassing different brain areas, creating a system for self-related cognitive activity (99). In healthy individuals, this network is normally active during internally oriented processes, and deactivates during external attention-driven cognitive tasks (94, 99, 100). In MDD, the DMN is known to be dysregulated and to display a decrease in task-related deactivation compared to HC that might contribute to the reduced response to external stimuli characteristic of MDD (74, 101–103). This characteristic might have been enhanced by the Go/No-Go task, which requires a shift of the subject's attention from internally-oriented thinking to external cues.

Our evidence of higher precuneus activity in adult-onset MDD patients compared to HC is concordant with the symptomatology of emotional and attentional dysregulation and with previous fMRI findings (104–106). Moreover, the abnormalities found in this area are also postulated to underline other specific characteristics of MDD, such as the negative representation of the self (107, 108) and rumination (109, 110). Thus, our results support the hypothesis that the DMN represents a putative biomarker of depression also in the adult-onset population, which can explain depressive symptoms like the ones that rely on self-referential processes.

Our sample of adult-onset MDD patients also showed higher fMRI responses to the “Go/No-Go” condition (vs. rest) in the right lingual gyrus. This area is known to regulate visual processing and is in close relation to the primary visual cortex (calcarine fissure) (111). In MDD, activity and connectivity alterations in this structure (108, 112) have been observed. Overall, these results support the involvement of this area in working memory and response inhibition, two key cognitive domains known to be impaired in depression and that are mostly tested during the “Go/No-Go” condition (113–116).

#### Diagnosis by Sex Modulation of fMRI Activations

An interaction between diagnosis and sex was observed in the fMRI activation patterns associated with the task and especially the inhibitory “Go/No-Go” condition. Indeed, opposite differences between sexes and groups were observed in the right calcarine cortex and the right parahippocampal gyrus. The fMRI response in these regions was more negative, reflecting a higher deactivation, in healthy males vs. healthy females and in adult-onset MDD females vs. adult-onset MDD males.

Notably, regarding the right calcarine cortex, a similar pattern of diagnosis by sex effects was observed on its regional GM volume, providing a neuroanatomical basis to the fMRI results. This region is housing the primary visual cortex (85) and has an important role in targeting attention (117). As already mentioned, sexual dimorphism in this area has been previously documented in healthy brains, supporting the existence of different visual processes in the two sexes depending on the task. The interactions with diagnosis may further suggest sex-specific attentional processes elicited by the Go/No-Go paradigm in MDD individuals, but this result needs to be confirmed on larger samples.

The opposite sexually dimorphic pattern observed in adult-onset MDD patients, with females showing a more negative response than males, might reflect the role of calcarine gyrus in MDD symptomatology. In literature a plethora of alterations have been documented (92, 93), and have been associated both to resistance to treatment (118) and pathological cognitive processes, such as self-isolation, social avoidance, and impaired attentional performances (119).

In light of this evidence, the more negative fMRI response that we observed in adult-onset MDD females can explain the presence of a more severe symptomatology, documented by higher HDRS scores, and to the worse performance in the Go/No-Go task, maybe due to attentional impairments, observed in this subgroup of participants compared to the male counterpart. This evidence, if confirmed on larger samples, lets us hypothesize a role of the calcarine cortex in the pathological cognitive models documented to be more frequent in females than in males also in adult-onset MDD (4, 13, 120).

With regards to the right parahippocampal gyrus, this region is part of the limbic system and is primarily involved in spatial memory and retrieval of associative contextual memory (121, 122). Interestingly, sexual dimorphism in this area in healthy brains, from both structural and functional perspectives, has been documented (25, 123). From the functional perspective, healthy males have been characterized by lower functional connectivity and higher efficiency than healthy females, possibly associated with higher flexibility and better adaptation to stress (124), but differences in the mechanisms of autobiographical memory retrieval have been proposed as well (125). In contrast, meta-analytic evidence supports an increased right-sided activation of this region during working memory tasks in females than in males (126).

Notably, an opposite sexually dimorphic pattern of parahippocampal activation was observed in adult-onset MDD patients, with females having a more negative response compared to males. Several strands of evidence reported the key role of the parahippocampal gyrus in the pathophysiology of depression. Indeed, the region is considered to be a component of the DMN (96, 127, 128) and deficits in this region have been associated with clinical symptoms often observed in MDD, including rumination, abnormal retrieval of autobiographical memory (109), and generation of contextual associates (129). In addition, there is evidence of its role in emotion-mediated memory formation (96, 128) and emotional regulation (128, 130, 131), possibly contributing to impairments in reward and in the development of happy feelings (108). Nevertheless, our preliminary evidence is new in the literature. If reproduced on larger samples, the parahippocampal deactivation that we observed in MDD females during response inhibition might either contribute to and/or result from the female-specific symptomatology (4, 13, 120) characterized by detrimentally high stress levels (4, 13, 75, 132).

### Limitations

The main limitation of our study resides in the small number of participants, which significantly limited the statistical power of the results, enabling the extraction of MDD and sex effects at a trend level. Despite our focus on middle and late adulthood, the large age range of the participants might have introduced aging-related confounding effects. Moreover, we did not perform diagnosis or sex by age analyses as well as connectivity analyses, which should be implemented in future replications. The reliability and specificity of our results might have also been affected by the patients' pharmacological therapy, which was characterized by diverse substances and dosages among subjects, and by the females' fertility phase and menstrual cycle, whose status was not assessed during the clinical evaluation. Finally, it should be noticed that our pilot evidence was obtained on a sample of patients with MDD onset during adulthood. While this choice represents the main asset of our study, future replications on larger independent samples and on subjects with earlier illness onset are highly encouraged to confirm and better characterize our results.

## Conclusions

Our pilot investigation of sex differences in brain morphology and in the neural bases of inhibitory control in adult-onset MDD showed promising results that, if confirmed, might underly the different disease presentations that have been extensively documented in the two sexes. Our findings strengthen the idea that sex is a relevant factor shaping the neurobiological correlates of depression, and can serve as an incentive to future investigations.

## Data Availability Statement

The raw data supporting the conclusions of this article will be made available by the authors, without undue reservation.

## Ethics Statement

The studies involving human participants were reviewed and approved by Comitato Etico Milano Area 2, Fondazione IRCCS Ca' Granda Ospedale Maggiore Policlinico, Milan. The patients/participants provided their written informed consent to participate in this study.

## Author Contributions

MP: formal analysis and writing–original draft. EM: conceptualization, data curation, methodology, software, formal analysis, and writing–original draft. GD: data curation and writing–original draft. AF and SP: data curation, supervision, and writing–review and editing. DG, EF, PE, CC, and FT: data curation and writing–review and editing. JS: methodology, software, and writing–review and editing. EB: conceptualization, supervision, and writing–review and editing. PB: conceptualization, project administration, supervision, and writing–review and editing. All authors contributed to the article and approved the submitted version.

## Funding

The experimental research work was partially supported by the Fondazione Cariplo (Grant no. 2016-0908). Grant support to the authors was also received by the Italian Ministry of Health (Grant no. 2018-12367789 to EM).

## Conflict of Interest

The authors declare that the research was conducted in the absence of any commercial or financial relationships that could be construed as a potential conflict of interest.

## Publisher's Note

All claims expressed in this article are solely those of the authors and do not necessarily represent those of their affiliated organizations, or those of the publisher, the editors and the reviewers. Any product that may be evaluated in this article, or claim that may be made by its manufacturer, is not guaranteed or endorsed by the publisher.
