# Supplementary material for: Sexual Dimorphism in the Brain Correlates of Adult-Onset Depression: A Pilot Structural and Functional 3T MRI Study
Source: Front Psychiatry. 2022 Jan 5;12:683912. doi: 10.3389/fpsyt.2021.683912 (PMC8766797; doi:10.3389/fpsyt.2021.683912)

Supplementary material

1. Supplementary results

The following section illustrates the group-level fMRI activations induced by the Go/No-Go task (all conditions) compared to rest and by the "Go/No-Go" condition (inhibition mode) with respect to the "Go" condition (excitation mode) after removal of diagnosis, age, and sex effects. The significant clusters ($p<0.05$, cluster-based family wise error corrected) are illustrated in Figure S1, and their anatomical localization and statistical information are reported in Table S1.

1.1 Task vs. rest

The task, considering both excitatory and inhibitory conditions, elicited the group-level activation of a big portion of the **left precentral gyrus** and of the **bilateral inferior frontal gyrus (opercular portions).** Positive BOLD responses to the task compared to rest were also observed in the **left middle frontal gyrus and left insular cortex,** and in the **bilateral vermis and cerebellum** (Figure S1, left panel).
Conversely, negative BOLD responses to the task compared to rest were observed in bilateral portions of **the calcarine cortex, middle occipital cortex, precuneus, cuneus, and lingual gyrus,** and in the **right hemisphere at the interface between precentral and postcentral gyri (Figure S1, central panel).**

1.2 "Go/No-Go" vs. "Go"

The group-level comparison between the inhibitory ("Go/No-Go" blocks) and excitatory ("Go" blocks) conditions and revealed a set of clusters with higher BOLD response in the inhibitory mode (Figure S1, right panel). This pattern was observed in bilateral portions of the precentral gyrus, inferior parietal cortex, fusiform gyrus, vermis, and cerebellum, as well as in clusters in the left thalamus, right frontal cortex (middle and superior portions), and right middle cingulate cortex.

2. Supplementary tables

Table S1. Task-related fMRI activations in the entire group.

Contrast	# voxels	x y z	AAL regions	T-stat	p (cFWE)
Task > rest	2633	**-40 -16 54**	Precentral gyrus, L	9.41	<0.001
	423	**56 12 10**	Inferior frontal gyrus, opercular R	8.26	<0.001
	152	**-40 -60 -30**	Cerebellum crus 1, L	7.37	<0.001
	143	**-30 42 20**	Middle frontal gyrus, L	8.12	<0.001
	171	**-22 8 26**	Caudate, L	7.14	<0.001
	129	**4 -56 -6**	Vermis 4 5	7.06	<0.001

	130	**-58 10 24**	Inferior frontal gyrus, opercular L	6.60	<0.001
	310	**36 -60 -26**	Cerebellum 6 R	6.56	<0.001
	62	**-46 4 2**	Insular gyrus L	5.57	0.007
	72	**-32 20 10**	Insular gyrus L	5.08	0.004
Task < rest	1389	**22 -96 -4**	Calcarine cortex R	12.92	<0.001
	1277	**-32 -86 -12**	Lingual gyrus, L	12.60	<0.001
	4181	**14 -60 14**	Calcarine cortex R	8.94	<0.001
	73	**36 -20 38**	Postcentral gyrus, R	5.66	0.014
	86	**-38 -80 22**	Middle occipital gyrus L	4.84	0.006
"Go/No-Go" > "Go" *(inhibition > excitation)*	190	36 -56 -26	Cerebellum 6, R	8.57	<0.001
	240	42 6 36	Precentral gyrus, R	7.72	<0.001
	141	38 -50 46	Inferior parietal gyrus, R	7.27	<0.001
	94	-40 0 52	Precentral gyrus, L	6.96	<0.001
	289	8 12 46	Middle cingulate gyrus, R	6.81	<0.001
	47	-14 -8 16	Thalamus, L	6.70	0.035
	47	-44 -48 52	Inferior parietal gyrus, L	6.52	0.035
	62	2 -72 -20	Vermis 6	6.30	0.008
	54	32 -78 -18	Cerebellum crus 1, R	6.05	0.017
	72	4 20 34	Middle cingulate gyrus, R	6.00	0.003
	73	42 50 14	Middle frontal gyrus, R	6.00	0.003
	59	22 2 58	Superior frontal gyrus, R	5.73	0.011
	71	-26 -74 -24	Cerebellum crus 1, L	5.62	0.004
	49	6 -70 54	Precuneus, R	5.60	0.028
	53	28 4 68	Superior frontal gyrus, R	5.52	0.019
	77	28 48 12	Superior frontal gyrus, R	5.19	0.002
	49	-32 -52 -22	Cerebellum 6, L	4.87	0.028

AAL: automated anatomical labeling atlas. T: T-statistics. p: p-value. c(FWE): cluster-based family-wise error correction. x y z: MNI coordinates expressed in mm. MDD: major depressive disorder. HC: healthy controls. L: left. R: right.

Table S2. Medication types and dosages in adult onset MDD.

Class	Type	Dosage
SSRIs	Fluoxetine	28 mg
	Escitalopram	5 mg
	Citalopram	20 mg
SNRIs	Duloxetine hydrochloride	60 mg
	Venlafaxine	75 mg
BDZ	Bromazepam	10 gtt
	Alprazolam	50 ml
	Flurazepam	15 mg
AC	Gabapentin	300 mg
Lithium	Carbolithium	Unknown
NSAIDs	Indomethacin	When Needed
AAP	Quetiapine	25 mg

MDD: major depressive disorder. SSRIs: selective serotonin reuptake inhibitors; SNRIs: serotonin and norepinephrine reuptake inhibitors; BDZ: benzodiazepines; AC: anticonvulsants; NSAIDs: non-steroidal anti-inflammatory drugs; AAP: atypical antipsychotics. * The data from one female adult onset MDD are not available.

Table S3. SCID-5 diagnostic categories in adult onset MDD.

Diagnosis	N, sex
MDD, single episode in partial remission	1 M
MDD, single episode, in complete remission	3 M
MDD, recurrent episode, in partial remission	1 F
MDD, active disease, mild single episode	1 M, 1 F
MDD, active disease, moderate single episode	1 M, 2 F
MDD, single episode, in complete remission, history of panic disorder	1 F
MDD, active disease, recurrent severe episode	1 M

F: female; MDD: major depressive disorder; M: male; SCID-5: structured clinical interview for DSM V.

Supplementary Figure 1. Group-level fMRI results. Brain clusters with significant positive and negative fMRI responses to the task (A. and B. panels, respectively) and higher fMRI response to the “Go/No-Go” inhibitory blocks compared to the “Go” excitatory blocks (C. panel) in the entire sample ($p<0.05$, cFWE corrected). The cluster colors represent the General Linear Model T-contrast statistics (ranging from 3 to 4).

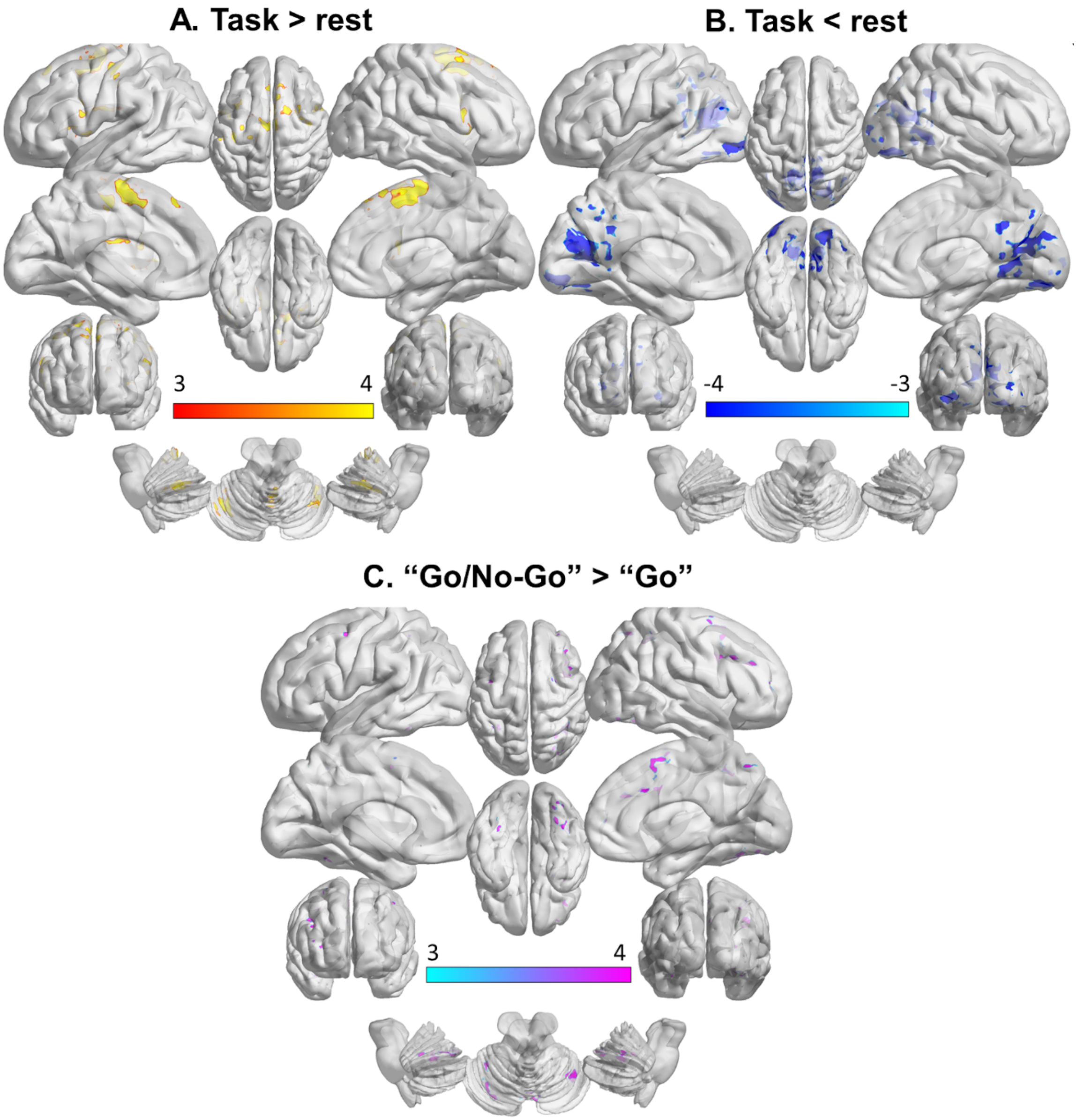

Supplementary F~~f~~igure 2. Go/No-Go task. This figure illustrates the Go/No-Go Task, composed ~~by~~of 16 trials of Go and No-Go blocks. The stimuli in each block are presented either at random or at fixed intervals of time.

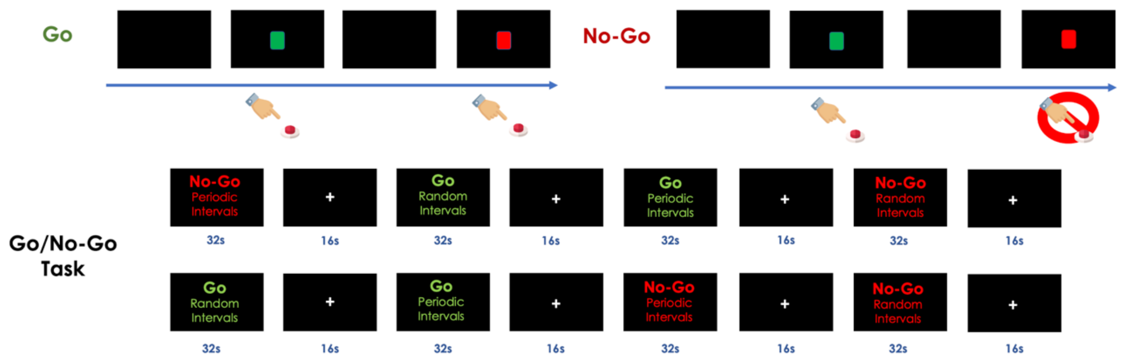

Supplement: Supplementary file 1 [file Data_Sheet_1.PDF]
